# Sodium Taurocholate Cotransporting Polypeptide (NTCP) Deficiency Hidden Behind Citrin Deficiency in Early Infancy: A Report of Three Cases

**DOI:** 10.3389/fgene.2019.01108

**Published:** 2019-11-07

**Authors:** Hui Lin, Jian-Wu Qiu, Yaqub-Muhammad Rauf, Gui-Zhi Lin, Rui Liu, Li-Jing Deng, Mei Deng, Yuan-Zong Song

**Affiliations:** Department of Pediatrics, The First Affiliated Hospital, Jinan University, Guangzhou, China

**Keywords:** cholestasis, citrin deficiency, sodium taurocholate cotransporting polypeptide deficiency, *SLC25A13*, *SLC10A1*, variant, child

## Abstract

Sodium taurocholate cotransporting polypeptide (NTCP), a carrier protein encoded by the gene *SLC10A1*, is expressed in the basolateral membrane of the hepatocyte to uptake bile acids from plasma. As a new inborn error of bile acid metabolism, NTCP deficiency remains far from being well understood in terms of the clinical and molecular features. Citrin deficiency is a well-known autosomal recessive disease arising from *SLC25A13* mutations, and in neonates or infants, this condition presents as transient intrahepatic cholestasis which usually resolves before 1 year of age. All the three patients in this paper exhibited cholestatic jaundice and elevated total bile acids in their early infancy, which were attributed to citrin deficiency by *SLC25A13* genetic analysis. In response to feeding with lactose-free and medium-chain triglycerides-enrich formula, their clinical and laboratory presentations disappeared gradually while the hypercholanemia persisted, even beyond 1 year of age. On subsequent *SLC10A1* analysis, they were all homozygous for the well-known pathogenic variant c.800C > T (p.Ser267Phe), and NTCP deficiency was thus definitely diagnosed. The findings in this paper indicated that NTCP deficiency could be covered up by citrin deficiency during early infancy; however, in citrin-deficient patients with intractable hypercholanemia following resolved cholestatic jaundice, NTCP deficiency should be taken into consideration.

## Background

Sodium taurocholate cotransporting polypeptide (NTCP) is a carrier protein in the basolateral membrane of the hepatocyte to uptake bile acids from plasma, playing a crucial role in the enterohepatic circulation of bile acids (Hagenbuch and Dawson, 2004). Although the causative gene *SLC10A1* was cloned as early as in 1994 (Hagenbuch and Meier, 1994) and NTCP function has been studied extensively (Ho et al., 2004; Pan et al., 2011; Yan et al., 2012; Yan et al., 2014), NTCP deficiency, as an inborn error of bile acid metabolism, was just described in very recent years. It was in 2015 that the first patient with NTCP deficiency was reported by Vaz et al. (2015). Following that, some articles involving patients with NTCP deficiency have been published (Deng et al., 2016; Liu et al., 2017; Qiu et al., 2017; Song and Deng, 2017; Van Herpe et al., 2017; Li et al., 2018), but the reported patients were rather limited in number, and the genotypic and phenotypic features of this condition remained far from being completely understood.

Citrin, a bipartite protein in the mitochondrial inner membrane, has been well-known as the aspartate-glutamate carrier isoform 2 (AGC2), playing a significant role in the malate shuttle, urea cycle as well as gluconeogenesis from lactate (Begum et al., 2002; Saheki et al., 2005; Palmieri, 2013; Palmieri, 2014). *SLC25A13*, the gene encoding citrin, was cloned in the year 1999 (Kobayashi et al., 1999), and citrin deficiency encompassed three age-dependent clinical phenotypes, i.e. Neonatal Intrahepatic Cholestasis caused by Citrin Deficiency (NICCD) in neonates or infants (Ohura et al., 2001; Tazawa et al., 2001; Tomomasa et al., 2001), adult-onset citrullinemia type II (CTLN2) in adolescents or adults (Kobayashi et al., 1999), and Failure to Thrive and Dyslipidemia caused by Citrin Deficiency (FTTDCD) at pediatric age beyond 1 year (Song et al., 2011; Saheki and Song., 2017). To the best of our knowledge, although the clinical and molecular characteristics of NICCD has been studied for years (Ohura et al., 2007; Chen et al., 2013; Song et al., 2013; Ricciuto and Buhas, 2014; Zeng et al., 2014; Wang et al., 2015; Lin et al., 2016; Zhang et al., 2017), patients with NTCP deficiency complicated by NICCD have never been reported thus far.

Very recently, our team diagnosed three pediatric patients suffering from citrin deficiency and NTCP deficiency as well, and their molecular and clinical findings were reported herein.

## Case Presentation

**Figure 1:**
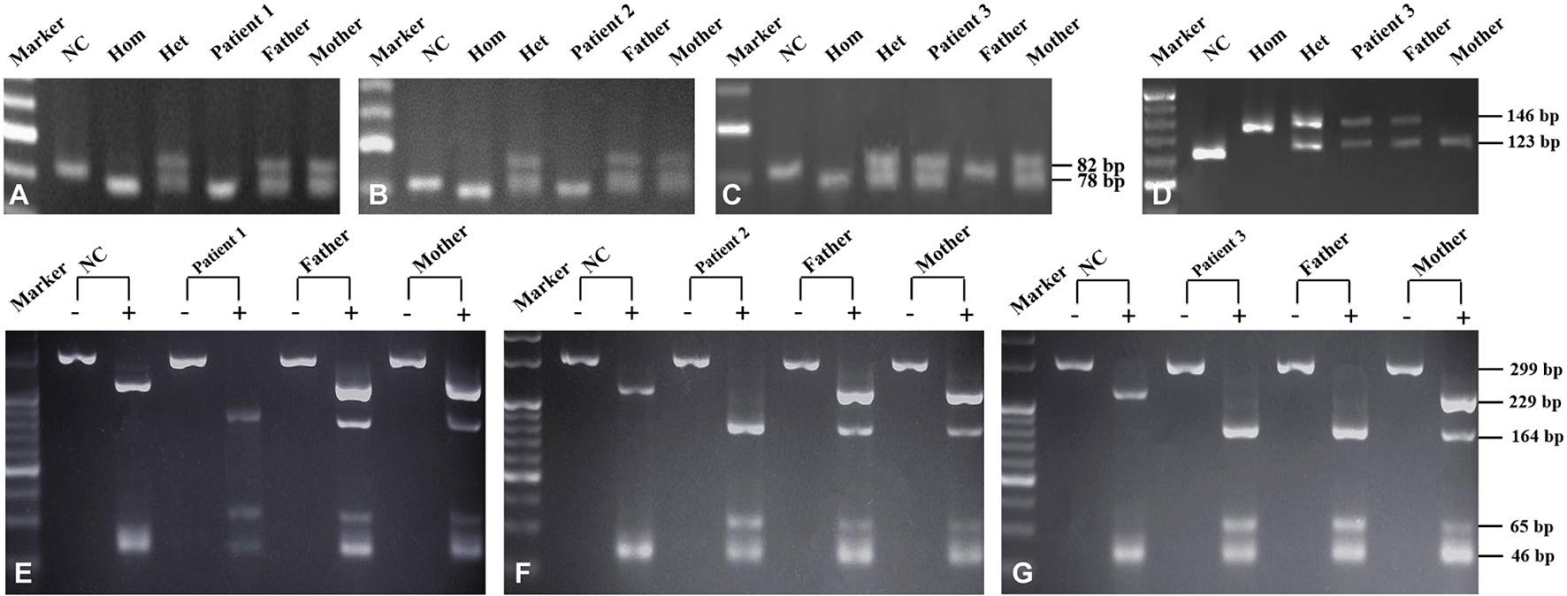
*SLC25A13* and *SLC10A1* genetic analysis of the three families. *SLC25A13* analysis revealed that patients 1 **(A)** and 2 **(B)** were both homozygotes of the c.852_855del4 mutation while patient 3 **(C** and **D)**, a compound heterozygote of the mutations c.852_855del4 and c.1638_1660dup. On *SLC10A1* analysis, patients 1 **(E)**, 2 **(F)**, and 3 **(G)** as well as her father were all homozygotes, while the parents of patients 1 and 2 and the mother of patient 3, all carriers, of the variant c.800C>T(p.Ser267Phe). NC (normal control), Hom (homozygous control), Het (heterozygous control). The “-” and “+” over every lane in panels **E**–**G** represented with and without enzymatic digestion by using the HphI enzyme, respectively.

*Patient 1* was a 5–year-and-11-month-old female referred to the First Affiliated Hospital, Jinan University due to abnormal liver function discovered for 5 years and 7 months. When aged 4 months, she was admitted to a hospital in Guangzhou due to jaundice for 3 months, where physical examination revealed an enlarged liver 4.0 cm below the right costal margin, and a liver function test revealed elevated serum levels of total bilirubin (TBIL), direct bilirubin (DBIL), indirect bilirubin (IBIL), alanine transaminase (ALT), aspartate transaminase (AST), γ-glutamyl transpeptidase (GGT), and alkaline phosphatase (ALP), indicating cholestatic jaundice (**Table 1**). Blood amino acid spectrum analysis by tandem mass spectrometry (MS-MS) revealed raised citrulline, methionine, arginine, and threonine, while large quantities of galactose, galactitol, galactonate, and 4-hydroxyphenyllactate (4HPL) were detected on urinary gas chromatography-mass spectrometry (GC-MS) analysis. Considering the above clinical and laboratory findings, NICCD was suspected, and breast-feeding was stopped while a lactose-free and medium-chain triglycerides (MCT)-enriched formula was introduced. When aged 4.9 months, *SLC25A13* genetic analysis in our hospital unveiled a homozygote of the c.852_855del4 mutation (**Figure 1A**) and the diagnosis of NICCD was hence made. Thereafter, besides feeding with the therapeutic formula, supplemental foods rich in protein were encouraged. As a result, her liver function indices got improved gradually and returned to normal by age 10.2 months. However, the hypercholanemia was refractory, with total bile acid (TBA) levels fluctuating from 27.6 µmol/L to 340.2 µmol/L (reference range: 0–10 µmol/L) (**Table 1**). After the age 2 years, the patient showed a fondness for foods rich in protein and fat while an aversion to carbohydrate-rich diets.

**Table 1:** Biochemical alterations over time in patient 1 and the parents.

Indices (reference range)	Ages of patient 1														Father	Mother
	4 M	4.9 M	6 M	8 M	10.2 M	1 Y 1 M	1 Y 4 M	1 Y 7 M	1 Y 10 M	2 Y 3 M	2 Y 8 M	3 Y 8 M	4 Y 8 M	5 Y 11 M		
ALT (5–40 U/L)	54	52	81	79	38	29	19	28	22	39	18	19	14	20	28	18
AST (5–40 U/L)	124	98	113	74	33	32	40	32	33	53	32	61	29	24	19	20
GGT (8–50 U/L)	150	138	42	23	19	14	—	10	14	13	10	13	11	12	30	12
ALP (40–500 U/L)	644	353	355	259	341	327	—	388	232	227	287	224	277	257	77	65
TP (60.0–83.0 g/L)	50.6	63.4	61.2	64.2	63.4	69.4	—	69	71.9	70.2	74.6	73.7	73.9	73.9	75.1	75.4
AIb (35.0–55.0 g/L)	38.2	43.0	47.4	48.4	47.6	49.6	—	47.2	47.7	43.5	49.7	50.7	48.1	49.5	46.1	40.6
GIb (20.0–30.0 g/L)	12.4	20.4	13.5	15.8	15.8	19.8	—	21.8	24.2	31.3	20.5	23.9	25.6	24.4	29	34.8
Tbil (2–19 µmol/L)	97.3	28.2	7.2	5	4.4	4.5	5.1	7.1	4.9	5.5	5.0	7.7	5.5	4.8	7.6	6.6
Dbil (0–6 µmol/L)	73.9	21.8	4.0	2.3	1.4	1.4	1.4	2.3	0.6	2.1	1.5	2.6	1.7	1.7	1.9	1.3
Ibil (2.56–20.9 µmol/L)	23.4	6.4	3.2	2.7	3.0	3.1	3.7	4.8	4.3	3.4	3.5	5.1	3.8	3.1	5.7	5.3
TBA (0–10 µmol/L)	—	340.2	174	135.2	132.8	123.2	82.1	109.3	165.4	111.1	105.8	173.6	27.6	48.7	3.2	3.7

ALT, alanine aminotransferase; AST, aspartate aminotransferase; GGT, γ-glutamyl transpeptidase; ALP, alkaline phosphatase; TP, total protein; Alb, albumin; Glb, globulin; Tbil, total bilirubin; Dbil, direct bilirubin; Ibil, indirect bilirubin; TBA, total bile acids;-, not tested. For the ages, Y represents years; M, months.

As the first product of a non-consanguineous couple, the child was delivered spontaneously at the gestational age of 38 weeks and 3 days after an uneventful pregnancy, with a birth weight of 3.0 kg and body length 50 cm. The parents were healthy, and there was no family history of any genetic or infectious diseases.

Physical examination revealed a body temperature (T) 36.5°C, heart rate (HR) 115 beats/min (bpm), respiratory rate (RR) 20 bpm, weight (WT) 18 kg, height 107.0 cm. No jaundice was observed in the skin and sclera. The lungs were clear. No murmurs or abnormal heart sounds were heard. There was no abdominal distention, and the liver and spleen were not enlarged. Physiological reflexes were normal and no pathological reflexes could be found on nervous system examination. The extremities were warm, and the distal perfusion was excellent.

Laboratory investigation showed otherwise normal biochemical indices but a TBA level of 48.7 µmol/L. In view of the intractable hypercholanemia, NTCP deficiency was highly suspected, and *SLC10A1* genetic analysis was performed. As a result, the patient was a homozygote, while the parents, carriers, of the reportedly pathogenic variant c.800C > T(p.Ser267Phe)(**Figure 1E**). NTCP deficiency was thus definitely diagnosed. No specific therapy was given but close clinic follow-up was underway.

*Patient 2* was a 1-year-and-1-month-old male visiting our clinic due to hypercholanemia discovered for 11 months. At the age 2 months, he was referred to a local hospital because of prolonged jaundice for about 1 month. On biochemistry analysis, elevated serum levels of AST, GGT, ALP, TBIL, DBIL, and IBIL, together with decreased level of albumin were detected, and notably, the TBA level reached 268.7 µmol/L (**Table 2**). Subsequent urinary GC-MS analysis detected elevated 4-hydroxyphenylpyruvate (4-HPPV) and 4HPL, while raised levels of citrulline, methionine, and threonine were detected on MS-MS analysis of blood sample. When aged 2.1 months, the infant undertook *SLC25A13* analysis in our hospital, and proved to be a homozygote of the c.852_855del4 mutation (**Figure 1B**), and the diagnosis of NICCD was thus made. Then breast-feeding was stopped and a lactose-free and MCT-enriched formula was given. Then his jaundice disappeared rapidly, and serum bilirubin levels returned to normal at his age 5.5 months (**Table 2**). However, the hypercholanemia persisted, even beyond 1 year of age (**Table 2**). No steatorrhea or acholic stool was observed during the course of the disease.

**Table 2:** Biochemical changes over time in patient 2 and the parents.

Indices (reference range)	Ages of patient 2							Father	Mother
	2 M	2.1 M	3.2 M	5.5 M	8.5 M	1 Y 1 M	1 Y 6 M		
ALT (5–40 U/L)	23	15	23	21	31	35	11.5	35	13
AST (5–40 U/L)	105	45	48	34	41	42	31.6	34	18
GGT (8–50 U/L)	281	281	162	18	16	16	18	24	10
ALP (20–220 U/L)	1,681	422	312	247	209	214	170.5	79	78
TP (60.0–83.0 g/L)	49.1	45.5	57.8	60.2	61.9	68	70.2	76.9	73.2
AIb (35.0–55.0 g/L)	32.4	30	40.4	46.3	46.2	45.6	43.7	46.1	43.1
GIb (20.0–30.0 g/L)	—	15.5	17.4	13.9	15.7	22.4	26.5	30.8	30.1
Tbil (2–19 µmol/L)	204.4	79.1	15.5	4.7	7.3	6.7	6.0	8.8	8.1
Dbil (0–6 µmol/L)	93.3	43.2	12.7	2.2	1.4	1.5	1.1	1.9	2.2
Ibil (2.56–20.9 µmol/L)	111.1	35.9	2.8	2.5	5.9	5.2	4.9	6.9	5.9
TBA (0–10 µmol/L)	268.7	110.8	233.8	143.3	208.7	234.5	148	31.5	78.8

Abbreviations as in **Table 1**.

As the first child of a non-consanguineous couple, the patient was delivered vaginally at the gestational age of 37 weeks and 4 days with the birth weight 2700 g. The Apgar score was 9 points at 1 min and 10 points at 5 min after umbilical ligation. Parents were both hepatitis B virus (HBV) carriers, who were apparently healthy but with slightly raised serum TBA levels (**Table 2**). Family history of any genetic diseases was denied.

Physical examination revealed a body T 36.6°C, weight 10.5 kg, HR 126 bpm, and RR 32 bpm. No jaundiced skin and sclera was observed. On auscultation, no abnormal sounds were heard on the lungs and heart. There was no abdominal distention, and the liver and spleen were non-palpable. Primitive reflexes were normal and pathological reﬂexes could not be found on nervous system examination.

Laboratory test at visiting revealed a serum TBA level of 234.5 µmol/L and otherwise normal indices. *SLC10A1* genetic analysis demonstrated that the patient was a homozygote, and his parents, carriers, of the variant c.800C > T (p.Ser267Phe) (**Figure 1F**). The diagnosis of NTCP deficiency was thus made. No specific therapy was given but clinic follow up was suggested. His serum TBA level was 148 µmol/L (**Table 2**) when aged 1 year and 6 months, and a fondness for low-carbohydrate and high-protein foods was noticed since the age of 1 year.

*Patient 3* was a 1-year-and-2-month-old female referred to our hospital because of abnormal liver function discovered for 12.7 months. At the age 1.3 months, she went through a liver function test because of prolonged jaundice for 1 month, which showed raised levels of AST, GGT, ALP, TBIL, DBIL, and IBIL (**Table 3**). When aged 1.8 months, her TBA level was found to be as high as 172.0 µmol/L besides the cholestatic alterations (**Table 3**), and the MS-MS analysis revealed increased levels of tyrosine, citrulline, and methionine while large quantities of urinary 4HPPV and 4HPL were detected on GC-MS analysis. NICCD was consequently suspected, and breast-feeding was stopped while a lactose-free and MCT-enriched formula was suggested. Following that, her cholestatic jaundice got alleviated rapidly and the laboratory alterations recovered to normal levels by the age 5 months, while the hypercholanemia was intractable, even beyond 1 year of age (**Table 3**).

**Table 3:** Biochemical indices over time in patient 3 and the parents.

Indices (reference range)	Ages of patient 3									Father	Mother
	1.3 M	1.8 M	5 M	7.2 M	1 Y 2 M	1 Y 10 M	2 Y	2 Y 8 M	3 Y 4 M		
ALT (5–40 U/L)	—	29.3	23	23	21.3	14.4	18	21.5	14.7	14	12
AST (5–40 U/L)	112	92.1	37	42	35	32.8	38	39.2	31.3	19	14
GGT (8–50 U/L)	299	284.6	47	20	11.8	11.6	14	15	15.6	34	14
ALP (20–220 U/L)	1,124	731.0	267.0	168.0	225.1	174.7	226	190.2	136.6	75	38
TP (60.0–83.0 g/L)	38.89	44.2	61.8	65.3	61.3	68.1	70.5	78.5	71	77.1	74.7
Alb (35.0–55.0 g/L)	26.6	32.5	40.2	40.5	44.7	43.7	47.4	50.1	47.2	48.6	48.2
Glb (20.0–30.0 g/L)	12.29	11.7	21.6	24.8	16.6	24.4	23.1	28.4	23.8	28.5	26.49
Tbil (2–19 µmol/L)	266.17	255.4	8.1	8.2	5.4	8.7	10.1	3.3	5.1	19.9	10.4
Dbil (0–6 µmol/L)	69.26	89.3	3.0	2.4	0.9	2.0	1.7	1.6	1.7	3.8	2.1
Ibil (2.56–20.9 µmol/L)	196.91	166.1	5.1	5.8	4.5	6.8	8.4	1.7	3.4	16.1	8.3
TBA (0–10 µmol/L)	—	172.0	94.0	72.0	50.9	19.7	10.7	36.1	25.6	21.1	1.5

Abbreviations as in **Table 1**.

As the first child of a non-consanguineous couple, the infant was delivered by cesarean section at the gestation age of 38 weeks and 2 days with the birth weight 2,750 g. Her father was clinically healthy with an elevated serum TBA level of 21.1 µmol/L (0–10 µmol/L), and her mother was physically and biochemically healthy (**Table 3**). There was no family history of any genetic diseases.

Physical examination at referral revealed a body weight 10.1 kg, length 80 cm and head circumference 46 cm. No jaundice was observed in the skin and sclera. Examinations of the heart, the lungs, the abdomen, and nervous system were all normal.

Biochemical test at referral revealed a TBA level 50.9 µmol/L with otherwise normal indices (**Table 3**). On genetic analysis, the patient was a compound heterozygote of the *SLC25A13* mutations c.852_855del4 and c.1638_1660dup, which was inherited from the father and mother, respectively (**Figures 1C, D**); moreover, the patient and her father were both homozygous for the *SLC10A1* variant c.800C > T (p.Ser267Phe), while her mother was a carrier (**Figure 1G**). Hence, citrin deficiency and NTCP deficiency were definitely diagnosed for the infant. No specific therapy was given, and his TBA level tended downward to 25.6 µmol/L at the age of 3 years and 4 months (**Table 3**), still remaining beyond the upper limit. The patient also had a fondness of protein-rich foods while an aversion to carbohydrate-rich foods from the age 1 year.

**Figure 2:**
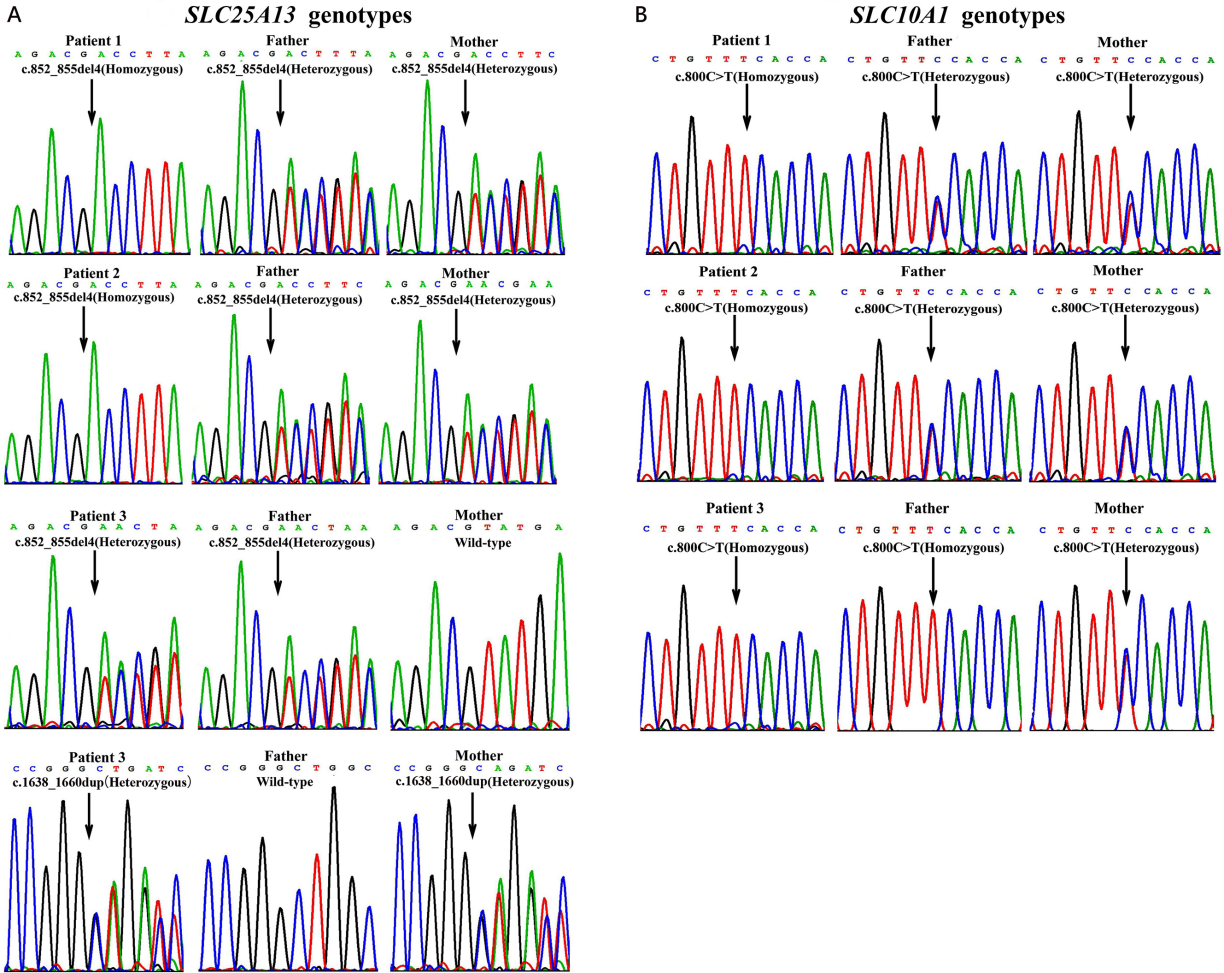
Genotyping results by Sanger sequencing analysis in the three unrelated families. On *SLC25A13* analysis **(A)**, patients 1 and 2 were both homozygotes of the c.852_855del4 mutation while patient 3, a compound heterozygote of the mutations c.852_855del4 and c.1638_1660dup. The parents of patients 1 and 2 as well as the father of patient 3 were all carriers of the c.852_855del4 mutation, while the mother of patient 3, a carrier of c.1638_1660dup. On *SLC10A1* analysis **(B)**, patients 1, 2, and 3 as well as her father were all homozygotes, while the parents of patients 1 and 2 and the mother of patient 3, all carriers, of the variant c.800C > T(p.Ser267Phe).

**Figure 3:**
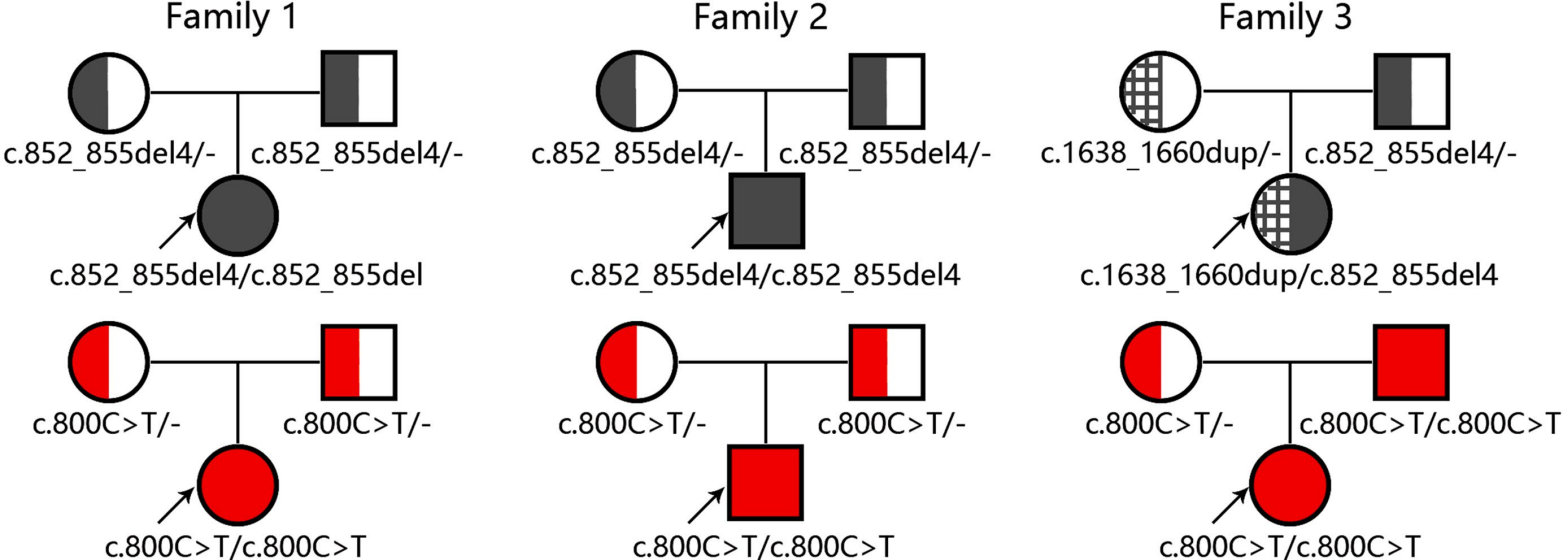
Diagrams for family trees with the causative genes *SLC25A13* and *SLC10A1*. The upper and lower panel illustrated the *SLC25A13* and *SLC10A1* genotyping findings in the three families, respectively.

The molecular findings above were further confirmed by Sanger sequencing (**Figure 2**) and illustrated as family tree diagrams (**Figure 3**). The clinical and molecular features of all the 3 patients were summarized in **Supplementary Table 1**.

## Discussion

All the three NICCD patients in this paper exhibited typical biochemical and clinical presentations of intrahepatic cholestasis, which were corrected by uptake of lactose-free and MCT-enriched formulas. Increased NADH/NAD^+^ ratio in the plasma of the hepatocyte was a critical pathophysiologic alteration of citrin deficiency, leading to energy shortage in the liver due to the impaired glycolysis (Saheki and Kobayashi, 2002; Saheki et al., 2010). MCTs were better absorbed as medium chain free fatty acids (MCFA) and transported *via* the portal vein, and then more quickly oxidized compared with long chain triglycerides (LCTs). MCFA oxidation within mitochondria produced acetyl-CoA, FADH2, and NADH to yield energy; and excess acetyl-CoA could enhance malate–citrate shuttle activity, generating more cytosolic NAD^+^, thus decreasing the NADH/NAD^+^ ratio (Hayasaka et al., 2012; Hayasaka and Numakura, 2018). On the other hand, the lactose was digested in the gut into glucose as well as galactose, and the latter was then absorbed into blood and conversed into glucose by way of Leloir pathway in the liver (Frey, 1996). The galactose metabolism in the hepatocyte increased cytosolic NADH/NAD^+^ ratio and inhibited the activity of uridine diphosphate (UDP)-galactose-4-epimerase (Maxwell, 1957; Saheki et al., 2002), and the resultant secondary galactosemia injured the hepatocyte and led to hepatic dysfunction (Ning et al., 2000; Bosch, 2006; Song et al., 2010). Therefore, the lactose-free and MCTs-enriched formulas exhibited therapeutic effectiveness in the NICCD patients in this paper. Moreover, the peculiar food preferences in the citrin-deficient children beyond NICCD stage might be a self-saving dietary behavior to avoid raising the NADH/NAD+ ratio by too much carbohydrate uptake (Saheki et al., 2004; Saheki et al., 2005; Saheki et al., 2008).

The prominent evidence suggestive of NTCP deficiency in the three patients was persistent hypercholanemia after age 1 year. As the major carrier protein in the enterohepatic circulation of bile salts, NTCP uptakes conjugated bile salts from the plasma compartment into the hepatocyte in a sodium-dependent way (Hagenbuch and Meier, 1994). The *SLC10A1* p.Ser267Phe variant has been proved to be pathogenic functionally, bioinformatically and clinically, rendering NTCP without any function to uptake bile acids (Ho et al., 2004; Yan et al., 2014; Deng et al., 2016; Liu et al., 2017). The impaired NTCP function might be partially compensated by other transporters to uptake bile acids from the plasma, such as Organic Anion Transporting Polypeptide (OATP) 1B1 and 1B3 in the basolateral membrane of hepatocytes; however, in the absence of NTCP, they could just played a limited role in bile acid clearance and were unable to compensate for loss of NTCP (Karpen and Dawson, 2015). As such, it was not surprising for the four patients with NTCP deficiency, including three children and the father in family 3, to present with refractory hypercholanemia in this study (**Tables 1**–**3**). It was noteworthy that, although hypercholanemia was the unique clinical presentation for NTCP deficiency, this biochemical change itself was just a nonpathognomonic marker suggestive cholestatic liver disease, making NTCP deficiency be covered up by NICCD at early infancy, as in the three pediatric patients reported in this study.

Although molecular techniques and genetic data have significantly improved the understanding of rare diseases in the recent years (Jia and Shi, 2017; Ni and Shi, 2017), it was rather rare for two genetic diseases of the liver to affect the same individual. In this paper, however, citrin deficiency and NTCP deficiency were found to affect three pediatric patients simultaneously. This rare finding might be explained by the relatively high prevalence of the two genetic conditions in south China, especially in Guangdong province where the three patients were located. The allele frequency of *SLC10A1* variant c.800C > T varied in different populations, with the highest incidence occurring in Southern China (8% and 12% in Chinese Han and Dai respectively), suggesting that this hypercholanemia affected 0.64% of the Southern Han as well as 1.44% of the Dai Chinese population (Liu et al., 2017). On the other hand, molecular epidemiological survey showed that the carrier rate of *SLC25A13* mutations was 1/940 in the north but 1/48 in the south of Yangtze River of mainland China (Lu et al., 2005). In particular, the carrier rate of five prevalent *SLC25A13* mutations (including c.851_854del, c.1638_1660dup, c.615+5G > A, IVS16ins3kb, and c.1399C > T) was about 1/47 in Guangdong province, with an estimated morbidity of 1/8,800 for patients with citrin deficiency (Zhang et al., 2014).

Interestingly, the parents of patient 2, two carriers of the p.Ser267Phe variant, also exhibited slightly elevated TBA levels (**Table 2**). However, this finding did not constitute a challenge against NTCP deficiency as an autosomal recessive disorder. As a reasonable explanation, their HBV carrier status might be responsible for their mild hypercholanemia. Actually, besides functioning as a carrier protein to uptake bile acids from plasma, NTCP had proven to be a functional receptor for HBV to cross the basolateral membrane, entering into the hepatocyte (Yan et al., 2012). It was reported that the NTCP residues between 157 to 165 were important for pre-S1 lipopeptide binding of the HBV large envelope protein, and contributed to HBV infections on HepG2 cells (Yan et al., 2012; Yan et al., 2013). Moreover, Yan et al. identified the HBV L-protein derived lipopeptides as inhibitors of NTCP, indicating that the specific pre-S1 lipopeptide binding might inhibit NTCP from transporting bile salts (Yan et al., 2014). In a word, being HBV carriers might block the function of NTCP to uptake bile acids from plasma.

In conclusion, this paper reported three pediatric patients with NTCP deficiency complicated by citrin deficiency. The findings indicated that NTCP deficiency could be covered up by citrin deficiency during early infancy; however, in citrin-deficient patients with intractable hypercholanemia following resolved cholestatic jaundice, NTCP deficiency should be taken into consideration.

## Data Availability Statement

All datasets generated and analyzed for this study are included in the article/**Supplementary Material**.

## Ethics Statement

This study has been approved by the Committee for Medical Ethics, the First Affiliated Hospital, Jinan University. The authors declare that this study was performed after written informed consent had been obtained from the parents of the three families, which permitted publication of this case report.

## Author Contributions

HL, Y-ZS, and RL performed data collection and drafted the initial manuscript. Y-ZS conceptualized and designed the study, critically reviewed and revised the manuscript. MD, L-JD, Y-MR, G-ZL, and J-WQ carried out the genetic analyses and reviewed the manuscript. Y-ZS managed and followed up the pediatric patients. All authors contributed to manuscript revision, read and approved the submitted version.

## Funding

The present study was supported by National Natural Science Foundation (NSFC) of China (Nos. 81570793, 81741080, and 81974057).

## Conflict of Interest

The authors declare that the research was conducted in the absence of any commercial or ﬁnancial relationships that could be construed as a potential conﬂict of interest.
