# Supplementary material for: Sodium Taurocholate Cotransporting Polypeptide (NTCP) Deficiency Hidden Behind Citrin Deficiency in Early Infancy: A Report of Three Cases
Source: Front Genet. 2019 Nov 7;10:1108. doi: 10.3389/fgene.2019.01108 (PMC6856633; doi:10.3389/fgene.2019.01108)
Supplement: Supplementary file 1 [file Table_1.docx]

## Case information

| Patient ID | Age | Gender | Family history | Gene | Country | SNV or SV | Disease name | Phenotype | Symptom |
| --- | --- | --- | --- | --- | --- | --- | --- | --- | --- |
| 1 | 5y | Female | No | SLC25A13, SLC10A1 | China | c.852_855del4(p.Arg284fsTer2), homozygous; c.800C>T(p.Ser267Phe),homozygous | Citrin deficiency; NTCP deficiency | NICCD | Cholestasis;  Hypercholanemia |
| 2 | 1y | Male | No | SLC25A13, SLC10A1 | China | c.852_855del4(p.Arg284fsTer2),homozygous; c.800C>T(p.Ser267Phe),homozygous | Citrin deficiency; NTCP deficiency | NICCD | Cholestasis;  Hypercholanemia |
| 3 | 1y | Female | No | SLC25A13, SLC10A1 | China | c.852_855del4 (p.Arg284fsTer2) and c.1638_1660dup  (p.Ala554fsTer16), heterozygous; c.800C>T(p.Ser267Phe),homozygous | Citrin deficiency; NTCP deficiency | NICCD | Cholestasis;  Hypercholanemia |

Family history: Yes, No or don’t know;

家族病史：是否有家族病史；

Country: The patient's nationality or the patient's race;

国家：病人的国籍或人种；

Mutation: Nucleotide and protein change information or genotype;

突变：核苷酸、蛋白质的改变或基因型信息；

Disease name: The exact disease the patient was diagnosed;

疾病名称：病人被确切诊断的疾病名称；

Phenotype: The phenotype of the disease;

表型：疾病的表型信息；

Symptom: The clinical symptom;

症状：临床症状信息

Example:

| Patient ID | Age | Gender | Family history | Gene | Country | Mutation/SV | Disease name | Phenotype (If unable to map to HPO, please list all the phenotypes | Symptom |
| --- | --- | --- | --- | --- | --- | --- | --- | --- | --- |
| 1 | 9 | Male | Don’t know | SOX2 | China | Chr3: g.181430628C>G, heterozygous NM_003106.3, NP_003097.1 c.480C>G, p.(Tyr160*) | Microphthalmia syndromic 3 | HP:0000568\|Microphthalmos; HP:0008538\|Sensorineural hearing impairment; HP:0001263\|Global developmental delay; HP:0006996\|Dysgenesis of corpus callosum | Syndromic; microphthalmia; SOX2 sequencing |
